# Challenges in implementing Indonesia's community-based chronic disease management program (Prolanis): A scoping review

**DOI:** 10.3934/publichealth.2025045

**Published:** 2025-09-11

**Authors:** Raden M. Febriyanti, Aalbrecht Alby Irawan, Nursanti Anggriani, Yudhie Andriyana, Rizky Abdulah

**Affiliations:** 1 Department of Biological Pharmacy, Faculty of Pharmacy, Universitas Padjadjaran, Jatinangor, West Java, Indonesia; 2 Doctoral Program in Mathematics, Faculty of Mathematics and Natural Science, Universitas Padjadjaran, Jatinangor, West Java, Indonesia; 3 Department of Mathematics, Faculty of Mathematics and Natural Science, Universitas Padjadjaran, Jatinangor, West Java, Indonesia; 4 Department of Statistics, Faculty of Mathematics and Natural Science, Universitas Padjadjaran, Jatinangor, West Java, Indonesia; 5 Department of Pharmacology and Clinical Pharmacy, Faculty of Pharmacy, Universitas Padjadjaran, Jatinangor, West Java, Indonesia

**Keywords:** chronic diseases, community-based intervention, non-communicable diseases, primary healthcare, Prolanis

## Abstract

Non-communicable diseases (NCDs) pose a major public health challenge worldwide, particularly in low- and middle-income countries (LMICs) like Indonesia, driven by urbanization, lifestyle changes, and environmental risks. Challenges such as constrained healthcare resources and socio-economic disparities hinder the effectiveness of NCD prevention and management. In response, Indonesia has implemented the Community-Based Chronic Disease Management Program (Prolanis), designed to promote regular monitoring, medication adherence, lifestyle modifications, and health education through primary health centers. This scoping review aimed to identify and map the barriers to Prolanis implementation across different regions and communities in Indonesia. A comprehensive literature search was performed in Scopus, ScienceDirect, and PubMed for peer-reviewed publications between 2014 and 2024. After the screening process, 38 peer-reviewed works met the inclusion criteria and were analyzed thematically. Thematic analysis indicated five major categories of barriers, including infrastructure and staffing constraints, low coverage, participation and adherence, socioeconomic and cost barriers, cultural and health literacy barriers, and pandemic-related disruptions. Key issues included inadequate human resources, inconsistent medical supplies, geographic barriers, patient time conflicts, and a lack of perceived benefit. Additionally, socio-economic challenges such as out-of-pocket expenses and transportation costs further restricted participation. Addressing these identified barriers is critical for improving the effectiveness of Prolanis and enhancing chronic disease management in Indonesia. These findings also contribute valuable insights for the implementation of community-based NCD programs in other LMIC settings.

## 1. Introduction

Non-communicable diseases (NCDs), including cardiovascular diseases, diabetes mellitus, chronic respiratory disorders, and cancer, are the leading cause of death, accounting for 75% of annual deaths. The 2019 Global Burden of Disease (GBD) study showed a continued rise in NCD prevalence, with a disproportionate impact in low- and middle-income countries (LMICs), which account for 73% of NCD deaths [1]. Limited healthcare resources, fragmented health systems, and socioeconomic disparities further constrain prevention and treatment in these settings [2],[3]. In Indonesia, this global pattern is compounded by a double burden of communicable and non-communicable diseases, with NCDs now the leading cause of mortality [4]. System capacity is challenged by shortages in the health workforce and constrained resources to address the growing NCDs [5]. Within the National Health Insurance (JKN), 43% of users presenting to hospitals have chronic multimorbidity [6]. Nationally, NCDs account for nearly three-quarters of all deaths, with cardiovascular diseases as the dominant driver [7].

Addressing NCDs requires an integrated effort that encompasses prevention, early detection, and optimization of health service [8],[9]. In many high-income countries (HICs), these components are embedded within mature primary care–led frameworks that operationalize longitudinal, team-based care. Prominent examples include the Chronic Care Model (CCM) and the Patient-Centered Medical Home (PCMH), both of which institutionalize proactive population, planned follow-up, clinical decision support within electronic medical records, self-management support, and linkages to community resources [10],[11]. HICs have also implemented structured disease-management programs and performance-linked primary-care contracts alongside national risk-factor screening [12],[13]. These frameworks are supported by robust primary healthcare infrastructure and well-established referral pathways, such as routine screening protocols, advanced diagnostic technologies, timely intervention mechanisms, and comprehensive insurance coverage, all collectively facilitating early detection and appropriate management [14].

However, these frameworks may not be directly transferable to LMICs without appropriate adaptation. Recent evidence highlights the lack of locally tailored strategies to monitor and manage risk factors, which continues to hamper efforts to detect and treat NCDs at earlier, more manageable stages [15]. Consequently, replicating models from high-income countries often has limited success in LMICs, where basic resources for diagnosis, follow-up care, and health promotion may be inadequate [9]. Recognizing these gaps, international health authorities and policymakers emphasize that each country needs to develop an approach tailored to its own conditions, leveraging existing infrastructure, cultural assets, and community networks [8].

Community-based interventions play a pivotal role in bridging gaps in chronic disease management by capitalizing on locally available resources, fostering social support, and enabling culturally sensitive health promotion [16]. A review of mathematical modeling studies on community-based interventions demonstrates their effectiveness, showing substantial reductions in diabetes prevalence, and emphasizes the critical role of culturally and contextually tailored strategies in the management of NCDs [17].

Evidence from several LMICs illustrates the effectiveness of such measures. India has implemented several community-based intervention measures to control NCDs, such as the National Tobacco Control Program and the National Program for Control of Diabetes, Stroke and Cardiovascular Diseases [18]. These initiatives have reportedly improved knowledge of NCD risk factors such as unhealthy diet, increased cholesterol, and overweight, and developed strategies to modify risk factors in diverse cultural and socioeconomic settings [18]. One lifestyle modification program in Brazil has also demonstrated positive outcomes in improving the community's knowledge of diabetes and enhancing the quality of life of diabetes patients [19].

In Indonesia, a key community-based intervention aimed at addressing NCDs is the Program Pengelolaan Penyakit Kronis (Prolanis). Launched in 2014 by the Indonesian Healthcare and Social Security Agency (BPJS Kesehatan), Prolanis is delivered through primary health centers and offers comprehensive services, including regular medical consultations, monthly medication refills, health education classes, biannual lab tests, home visits, Short Message Services (SMS) reminders, and group exercise sessions [20],[21]. The goal is to proactively engage patients in managing chronic illness, improving the quality of life, and preventing complications in a cost-effective manner [22]. Early evaluations of Prolanis have indicated favorable outcomes, including improved glycemic control in type 2 diabetes patients, reduced blood pressure in hypertension, enhanced patient self-management, and positive impacts on overall well-being [22],[23]. Despite its potential, the implementation of Prolanis has yet to achieve optimal effectiveness. Previous studies have identified several factors influencing medication adherence among Prolanis participants, including knowledge gaps, inconsistent family support, and a lack of standardized operational guidelines [23],[24]. Other studies revealed that Prolanis' implementation in Indonesia remains suboptimal due to various barriers, i.e., resource limitations, personnel availability, funding shortages, and the absence of standard operating procedures [21],[22].

While many barriers resemble those reported across LMICs, several features create a distinctly Indonesian profile. Indonesia's archipelagic geography and decentralized primary-care delivery generate pronounced inter-district variability in infrastructure and staffing, complicating equitable rollout from urban PHCs to rural or mountainous areas [25]–[27]. Prolanis' group exercise and biannual laboratory checks depend on local capacity for space, supplies, and point-of-care HbA1c testing, which are inconsistently available [27],[28]. Communication relies largely on one-way SMS, limiting two-way scheduling and follow-up in populations with variable digital access [27]. Cultural pluralism and persistent use of traditional remedies can displace clinic-based education and medication adherence [29],[30], and disaster-prone settings have periodically disrupted attendance and monitoring [31]. Finally, BPJS capitation and reporting requirements create administrative load at the PHC level, with heterogeneity in pharmacist availability and multidisciplinary staffing affecting fidelity to counselling and follow-up [32],[33]. These Indonesia-specific conditions shape barriers that differ in emphasis from those seen in broader LMIC syntheses and therefore warrant a country-focused evidence map.

Given the potential of Prolanis and the diverse challenges it faces, we conducted a scoping review to compile and systematically map evidence on implementation barriers. A scoping approach is appropriate due to methodological and contextual heterogeneity in the literature, which includes qualitative studies, cross-sectional surveys, quasi-experimental designs, and small cohort analyses. Reported outcomes vary widely, encompassing program coverage, participation rates, HbA1c testing frequency, supply chain reliability, human resources, and patient experiences across different settings. Under these conditions, our objective is to map the scope and nature of available evidence, clarify key program components, and identify gaps, rather than to pool effect sizes or grade comparative effectiveness.

Furthermore, to ensure policy utility beyond generic LMIC challenges, we structure our findings by Prolanis' design elements in each barrier. This yields actionable levers for program redesign, including standardizing SOPs and staffing norms for Prolanis sessions, establishing supply-chain KPIs for HbA1c kits and essential medicines, redesigning appointment systems, outreaching to hard-to-reach areas, and monitoring indicators centered on attendance and test-completion rates instead of only clinical outcomes. By linking barrier themes to these levers, this synthesis informs BPJS and PHC resource allocation and operational policy.

## 2. Materials and methods

This review was designed and reported in accordance with the PRISMA-ScR guidance [34]. The complete, reproducible database strategies are provided in Supplementary File S1. Briefly, we searched PubMed, Scopus, and ScienceDirect for peer-reviewed studies from 2014 to 2024, using program-specific terms (“PROLANIS”, “Program Pengelolaan Penyakit Kronis”).

### 2.1. Eligibility criteria

We included peer-reviewed empirical studies conducted in Indonesia that evaluated Prolanis delivery or any of its components within PHCs. Eligible designs comprised quantitative, qualitative, and mixed-methods studies. We included studies reporting implementation, coverage, attendance, adherence, resources and workflow, supply chain and laboratory availability, human resources, costs, cultural and health-literacy factors, and COVID-19 disruptions. We excluded non-primary sources, e.g., systematic reviews, editorials, commentaries, grey literature, studies not on Prolanis, and reports in which relevant outcomes could not be disaggregated.

### 2.2. Study selection

**Figure 1.**
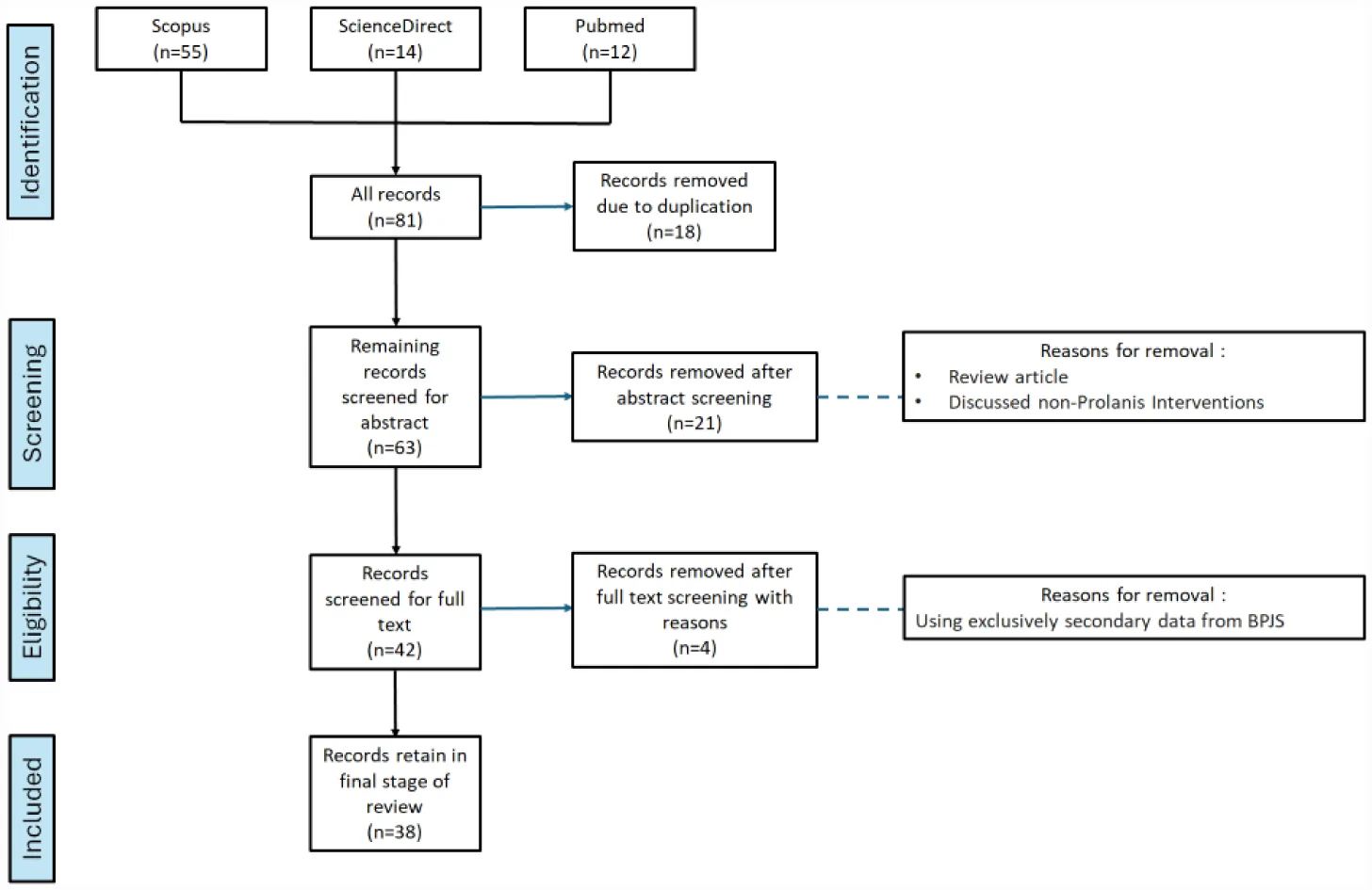
PRISMA flowchart illustrating the selection process for studies included in this review.

Search results were exported to Mendeley Reference Manager for automatic and manual de-duplication. Two reviewers (RMF, AAI) independently screened titles and abstracts and then full texts against the prespecified criteria; disagreements were resolved by consensus. Reasons for full-text exclusion were recorded. The selection process is summarized in the PRISMA-ScR flow diagram (Figure 1).

### 2.3. Data charting

We used a piloted data-charting form in accordance with our research questions (Table 1). RMF extracted and AAI verified all fields. Charted items included bibliographic details, province and region, urban or rural setting, study design and sample, and target condition (type 2 diabetes, hypertension, or both), which were mapped to five themes: infrastructure/staffing (I/S); low coverage/participation/adherence (LCPA); socioeconomic/cost (S/C); cultural/health literacy (C/HL); and pandemic-related (Pand).

### 2.4. Synthesis and analysis

Consistent with the scoping-review methodology as reported in Supplementary File S2 no formal risk-of-bias appraisal was undertaken; instead, we summarized the study design, setting, and sample characteristics to aid the interpretation of heterogeneity. We conducted an inductive thematic analysis to identify and refine recurrent barrier categories, with iterative consensus resolution for ambiguous cases. In addition, keyword co-occurrence mapping of included articles was performed using VOSviewer (v1.6.20) with the following parameters: analysis type = co-occurrence; unit = keywords; counting method = full counting; minimum occurrences per keyword = 3; layout = VOS mapping with modularity-based clustering; visualizations = density and network maps [35].

**Table 1.** Research questions.

**Questions**	**Purposes**
When were the data collected?	Prolanis was initiated in 2014, suggesting that initial challenges may have been greater compared to subsequent years. Understanding the data collection period provides context.
From which geographic location do the data originate?	Knowing the geographic source aids in understanding the intervention's context, considering economic and cultural influences.
What study design and methodology were employed?	The study's design and methods are important for evaluating its reliability and the applicability of its findings.
What quantitative results were obtained?	Numerical data analysis reveals relationships, trends, or patterns, assessing the study's strength and relevance.
What are the main findings?	Analyzing the results helps identify and categorize factors that hinder the intervention's effectiveness.

## 3. Results

After removing 18 duplicates, 63 articles underwent title and abstract screening, resulting in the exclusion of 25 papers for failing to meet the inclusion criteria, which is primarily due to the lack of primary data or addressing non-Prolanis programs. 38 studies remained for final inclusion (Figure 1).

**Figure 2.**
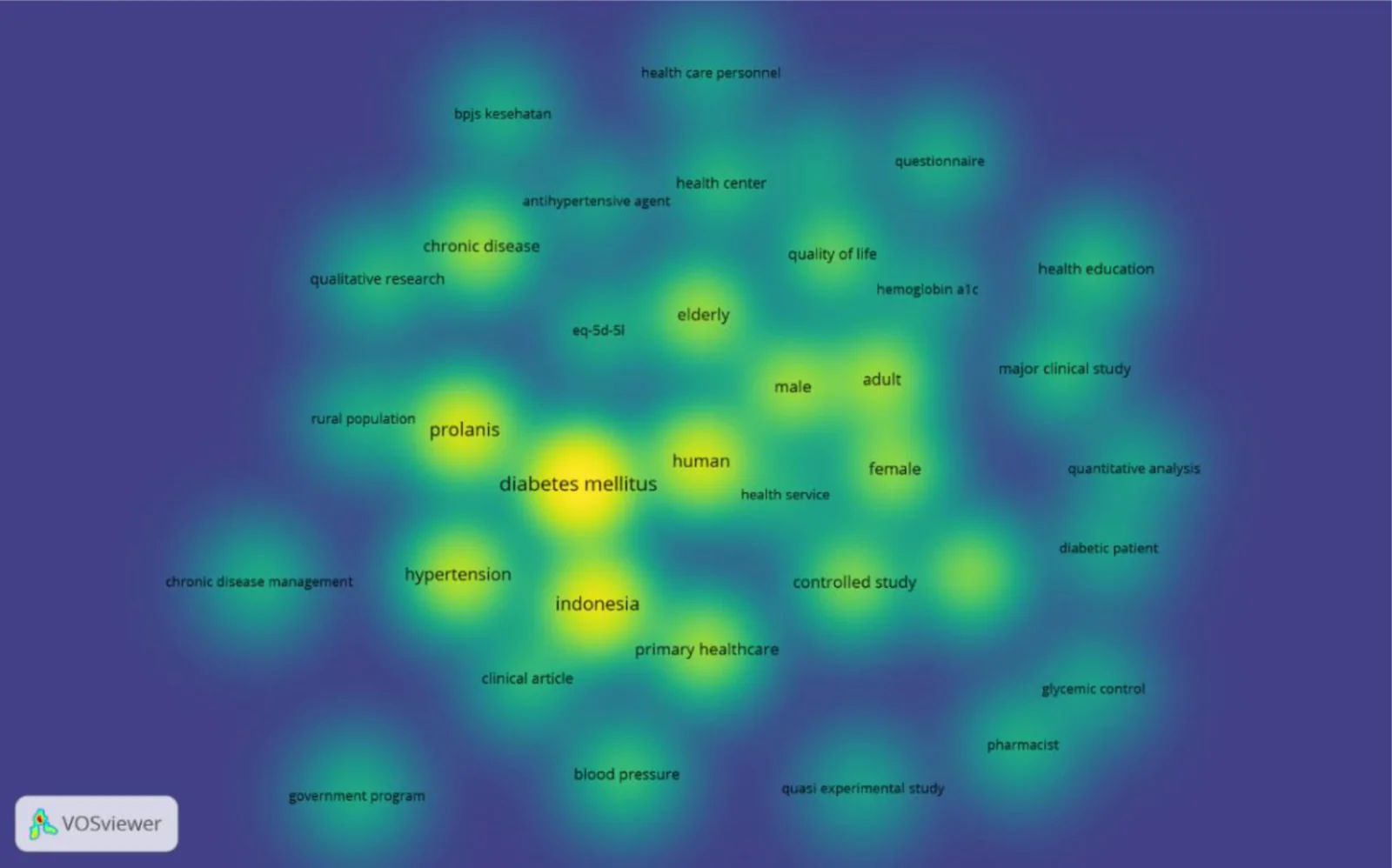
Keyword co-occurrence density map (VOSviewer).

Figure 2 presents a keyword co-occurrence density map in which nodes represent keywords and node size scales with frequency. Warmer color intensity reflects more frequent co-occurrence, proximity indicates stronger relatedness, and edges are suppressed for readability. In this map, “diabetes mellitus” (n = 32), “Prolanis” (n = 19), and “hypertension” (n = 15) form the principal hotspot, indicating that diabetes and hypertension dominate discussions of Prolanis implementation. Surrounding hotspots reflect the service platform (“primary healthcare”, “health care personnel”, “health center”, “bpjs kesehatan”), measurement (“HbA1c”, “quality of life”, “blood pressure”, “glycemic control”), and study methods (“questionnaire”, “quasi experimental study” “qualitative research”). Notably, references to specific Prolanis components, such as home visits, SMS reminders, or biannual laboratory checks, occur comparatively less often, implying that physical activity remains the most widely discussed intervention among the Prolanis services, with its role clearer in the network view.

**Figure 3.**
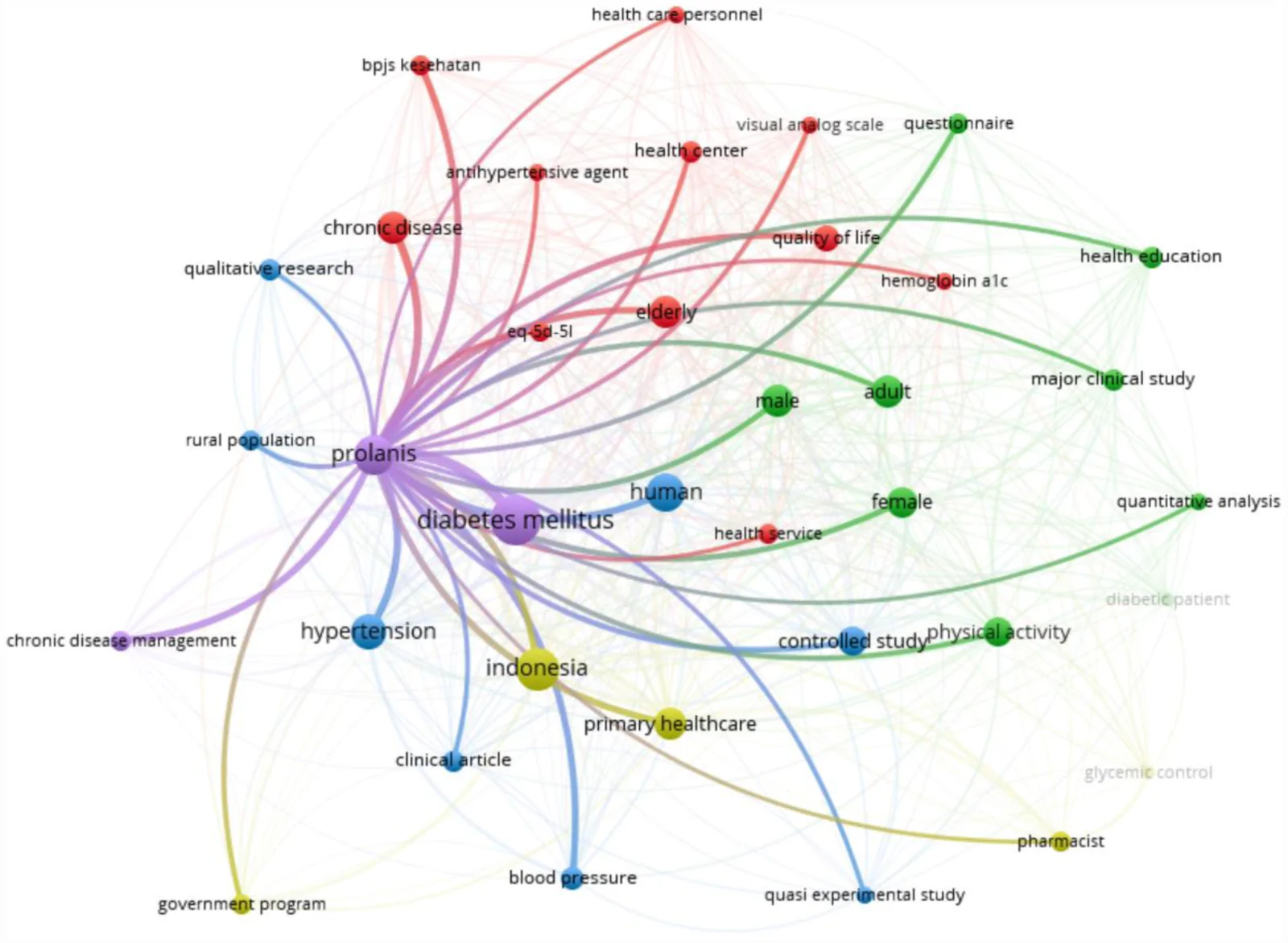
Keyword co-occurrence network (VOSviewer).

Figure 3 shows the same high-frequency nodes organized into modularity-based clusters with edges scaled by co-occurrence strength. These mirror the barrier themes discussed next: infrastructure and staffing, participation and adherence, socioeconomic and cost factors, and cultural and health literacy.

The 38 included articles (Table 2) span various research designs and geographical areas in Indonesia. Approximately half (n = 18) employed cross-sectional approaches, focusing primarily on describing barriers to Prolanis uptake or patterns of utilization. Purely qualitative studies (n = 6) used interviews and focus groups to explore sociocultural aspects, staff perceptions, and patient experiences. Additionally, six quasi-experimental evaluations and four retrospective cohorts assessed the effects of specific interventions, such as pharmacist-led counselling or structured exercise, on patient outcomes. One prospective observational study, one cluster-randomized trial, and one mixed-method pilot provided further insights into how Prolanis components may be optimized (Table 3).

**Figure 4.**
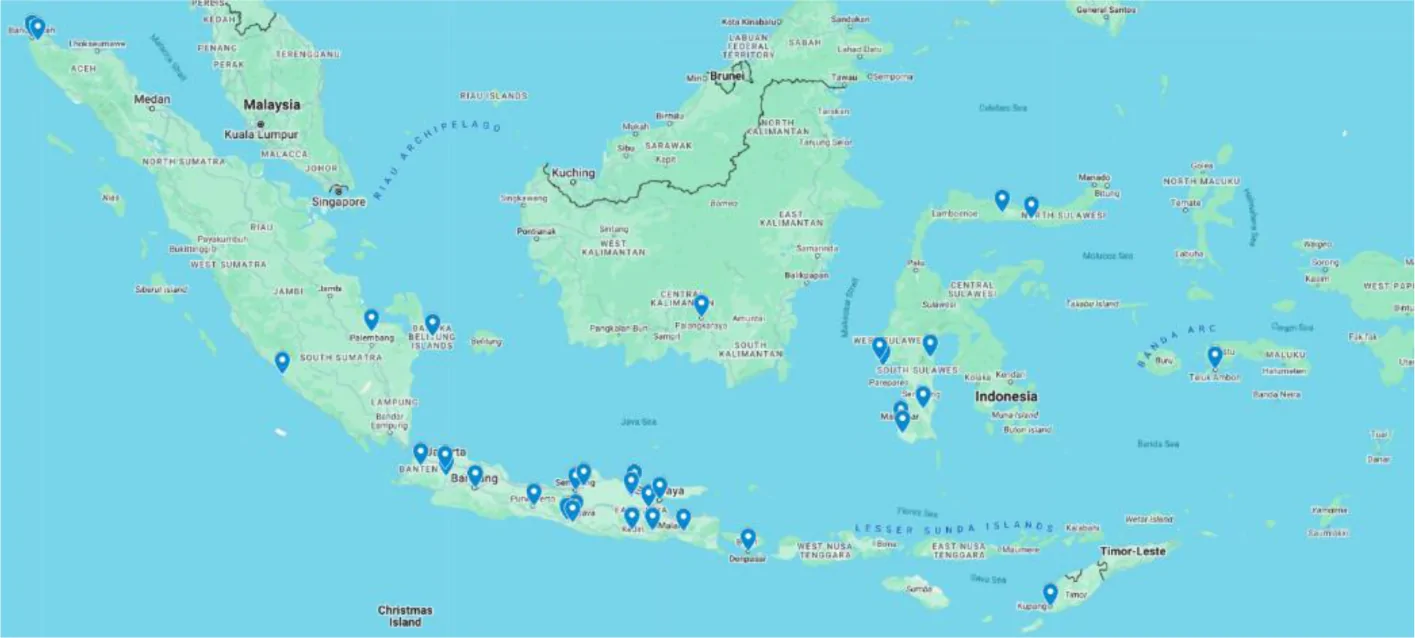
Location map of the study site.

Most studies (n = 29) were conducted in the provinces of Java, namely Yogyakarta, Central Java, East Java, West Java, and Banten, reflecting the island's higher density of healthcare facilities and academic institutions. Meanwhile, nine studies originated from other regions in Indonesia (South Sulawesi, West Sulawesi, Maluku, and Aceh), suggesting growing attention to Prolanis implementation in more remote or under-resourced regions (Figure 4). The included articles thus span diverse contexts, from urban Primary Health Centers (PHCs) to rural and mountainous districts.

The sample populations also varied, with some studies focusing on type 2 diabetes mellitus (T2DM), others on hypertension, and a smaller subset involving both conditions. Among the 14 intervention-oriented studies, comparison arms often included standard Prolanis care vs. an enhanced intervention, i.e., pharmacist-led counselling or structured exercise. Observational designs typically examined medication adherence, barriers to participation, and patient-reported outcomes. Several recurring themes emerged in relation to the broad research questions. First, infrastructure and staffing constraints consistently limited Prolanis rollout, particularly in rural or underserved areas [26],[36]. Second, low coverage, patient adherence, and high attrition were attributed to geographic inaccessibility, scheduling conflicts, and a lack of perceived benefit [24],[37]–[39]. Third, socioeconomic and cost barriers played a significant role in deterring regular participation, with out-of-pocket expenditures and transport fees frequently highlighted [40]. Fourth, cultural and health literacy challenges, such as reliance on herbal or traditional treatments and insufficient knowledge of healthy lifestyle practices, further weakened patient engagement [29],[41],[42]. Finally, pandemic-related disruptions reduced clinic visits and essential follow-ups, often exacerbating the progression of diabetes or hypertension [43],[44].

**Table 2.** Summary of the studies.

Author	Data collection	Study location	Study methodology	Sample/respondent	Key findings
Rahmawati et al. [36]	Aug–Nov 2015	8 rural villages, Bantul, DIY	XS; researcher-administered questionnaire; desc and inferential	N = 384 hypertension patients	Staff shortages; unclear roles; weak rural infra; multiple visits for meds; drug stock-outs
Sulistyaningrum et al. [40]	Jan–Dec 2016	16 PHCs in DIY (4 districts)	XS Obs; prescription records; descriptive cost analysis	N = 293 hypertension and/or T2DM patients	Higher drug costs among T2DM or combined T2DM + hypertension; significantly increased per-prescription cost; budget limits Prolanis coverage
Mubarak et al. [26]	Nov–Dec 2023	2 PHCs (Totoli, Sendana), West Sulawesi	Qual; purposive sampling	N = 4 key informants, 9 routine informants	HR variability across PHCs; poor governance; weak SOP compliance
Rokhmad et al. [37]	Apr–Jul 2022	3 PHCs, Tulungagung, East Java	XS; monthly NCD reports; descriptive + correlation	N = 546 T2DM patients, 187 in Prolanis, 359 not in Prolanis	Low rural participation; access/transport barriers; poor adherence; limited infra/staff; weak follow-up
Fajriani et al. [27]	Jan–Feb 2024	Biru PHC, Bone, South Sulawesi	Qual descriptive; IDIs, observation, docs; content analysis	N = 6 key informants; 3 Prolanis participants	Insufficient supplies/equipment; space/scheduling constraints; inconsistent labs; low smartphone access; budget constraints
Iskandarsyah et al. [45]	Aug 2017–Jan 2018	4 PHCs, Makassar, South Sulawesi	XS; EQ-5D-5L; utility index	N = 220 Prolanis participants with T2DM	Meds-only attendance; limited counselling time; low program awareness; low family support/adherence
Aungsuroch et al. [29]	Apr 2018	3 PHCs, Belitung, Bangka Belitung	Qual descriptive; phenomenology; FGD; content analysis	N = 20 Prolanis participants with hypertension	Dietary nonadherence (salt); preference for traditional remedies; self-medication; dosing confusion; transport barriers; limited education/home visits
Putri et al. [46]	Oct–Nov 2017	4 PHCs, DIY and Central Java	Qual; semi-structured interviews; TFA content analysis	N = 14 key informants	Unclear clinical impact; admin burden; low provider incentives; limited resources/awareness
Tanjung et al. [28]	May–Jul 2019	3 PHCs, Bandung Regency, West Java	Prospective Obs; CER on metformin and glimepiride	N = 60 Prolanis participants with T2DM	Mixed cost-effectiveness; follow-up challenges; resource constraints; variable cost components
Handayani et al. [30]	2019–2020	Semarang (Central Java) and Gorontalo City	XS; questionnaire; t-test/correlation	N = 294 T2DM patients	Context differences (Java vs. outside Java); unhealthy diet/herbal reliance; limited patient education
Khusna et al. [41]	Oct–Nov 2018	Depok Sleman PHC, DIY	Descriptive correlational XS; PDAQ	N = 85 Prolanis participants with T2DM	Poor diet adherence; nutrition knowledge gaps; limited counselling
Azam et al. [43]	2022	3 PHCs, Demak, Central Java	Observational	N = 164 Prolanis participants with T2DM	COVID worsened glycemic control; missed visits; med access barriers; less physical activity
Paradise et al. [47]	Dec 2023–Feb 2024	Kassi-Kassi & Kalukubodoa PHCs, Makassar	Prospective QE; nutrition/medication/PA interventions	N = 351 T2DM patients	Multicomponent adherence problems; staff shortages/monitoring gaps; need education + supervised exercise
Yusransyah et al. [48]	Jun–Aug 2019	16 PHCs, Pandeglang, Banten	QE; pharmacist-led counselling; MARS scale	N = 96 Prolanis participants with hypertension	Pharmacist shortages; symptom-driven nonadherence; low health literacy; resource constraints
Misnaniarti et al. [49]	2018	4 PHCs, Banyuasin, South Sumatra	XS; WHOQOL-BREF	N = 250 (142 in Prolanis 108 not in Prolanis)	Low coverage (~57%); irregular attendance
Armawati et al. [39]	Apr–Jun 2018	Makassar, South Sulawesi	QE; 3 intervention arms	N = 60 Prolanis participants with hypertension	Scheduling/compliance/staff limits
Alkaff et al. [50]	Apr 2018–Oct 2019	Wates PHC, Mojokerto, East Java	Observational retrospective cohort	N = 44 Prolanis participants with hypertension	BP control only; low attendance; staff shortages; refill-only visits
Sari et al. [24]	2021	12 PHCs, Bandung City, West Java	Correlational XS; HBM (46 items)	N = 235 Prolanis participants with T2DM	Mixed perceived benefits; scheduling/staff barriers; low attendance coverage
Yudha et al. [51]	Aug–Oct 2021	Denpasar City, Bali	XS; Zung Anxiety Scale	N = 384 Prolanis participants	Mild anxiety common; mental health gaps
Rofi'i et al. [42]	Mar–Oct 2020	Several PHCs, Tuban, East Java	XS; questionnaire + IPAQ	N = 105 T2DM patients	Low activity tied to knowledge/self-efficacy; infrastructure and family support gaps; exercise confusion
Souhaly et al. [31]	Early 2020	Lateri PHC, Ambon, Maluku	Analytic survey XS; interviews, questionnaires, records	N = 69 Prolanis participants	Disaster disrupted attendance; need psychosocial support
Zainuddin et al. [52]	Apr–Sep 2022	2 PHCs, Takalar, South Sulawesi	QE; HL + brisk walking vs. standard; HbA1c, IPAQ	N = 60 T2DM patients	Digital access barriers; staff shortages
Soleman et al. [53]	2019	25 PHCs, Sleman, DIY	XS; SERVQUAL (gap analysis, CSI, IPA)	N = 230 Hypertension or T2DM patients	Service gaps across dimensions; biggest in tangibility/empathy/reliability
Sholihat et al. [54]	Apr–Jun 2018	6 PHCs, Purwokerto, Central Java	XS; EQ-5D-5L; utility index + descriptive	N = 616 total (267 T2DM patients, 349 hypertension patients)	High pain/discomfort; notable anxiety/depression; need pain/mental health focus
Sholikatin et al. [55]	July 2017	Mojo PHC, Surabaya, East Java	XS correlation; multiple questionnaires	N = 24 Prolanis participants with hypertension	Low attendance; competing obligations; diet confusion; low health literacy
Iskandarsyah et al. [32]	Aug 2017–Aug 2018	4 PHCs, Makassar, South Sulawesi	Cluster RCT; monthly pharmacist counselling; EQ-5D-5L, HbA1c	N = 220 T2DM patients	Scheduling/attendance constraints
Salamah et al. [44]	Dec 2019–Dec 2020	3 PHCs, rural East Java	Pilot retrospective cohort; metabolic & renal parameters	N = 52 T2DM patients	COVID disrupted control; fewer visits; limited telemedicine
Krisnadewi et al. [33]	2024	5 regions (DIY, Denpasar, Boalemo, Palangkaraya, Kupang)	Qual descriptive; CFIR framework	N = 19 health workers	Implementation heterogeneity; infra/staff/budget/culture constraints; rural outreach difficult
Yuniartika et al. [56]	Apr 2019	PHC Kartasura, Sukoharjo, Central Java	QE; booklet+lecture vs. lecture-only; 20-item questionnaire	N = 40 Prolanis participants with T2DM	Education materials limited; education tailoring needed
Yusransyah et al.[38]	Jun–Aug 2019	16 PHCs, Pandeglang, Banten	XS; EQ-5D-5L (QoL)	N = 96 Hypertension patients	Resource/scheduling constraints
Salamah et al. [22]	Dec 2019–Dec 2020	4 PHCs, East Java	Retrospective observational; BMI, BP, lipids, eGFR (T0/T1/T2)	N = 91 Hypertension patients	COVID disrupted HT care; fewer visits; med/screening gaps
Kusumawardana et al. [57]	Oct–Nov 2021	6 PHCs, Bogor City, West Java	Quant survey XS; CSI-29 + IPM	N = 104 Prolanis participants	Moderate satisfaction (~75%); process/cost deficits; improve tangibility/responsiveness/empathy
Rahmawati et al. [25]	Oct–Nov 2016	Rural Bantul, DIY	Qual (IDIs+FGDs); thematic analysis	N = 13 health workers; 12 community health workers; 12 patients	Low health literacy/staff time; traditional medicine; lack of guidelines/synergy
Febriawati et al. [58]	2021–2022	Several PHCs, Bengkulu City	XS with correlation/regression	N = 211 Prolanis participants with T2DM	Attendance/resource gaps
Alkaff et al. [59]	Apr 2018–Oct 2019	Wates, Mojokerto, East Java	Retrospective cohort; metabolic parameters (6-mo)	N = 30 Prolanis participants with T2DM	Limited metabolic gains; staffing/follow-up/education gaps
Said et al. [60]	Late 2023–early 2024	Depok Jaya PHC, West Java	XS; correlation; program compliance + self-efficacy	N = 30 Prolanis participants with hypertension	Attendance/time/staff follow-up barriers
Sofyan et al. [61]	Baseline Data: Mar–Apr 2019; Interviews/FGDs: 2019–2020	Banda Aceh and Aceh Besar, Aceh	Mixed; descriptive stats + thematic analysis	N = 521 T2DM patients; 8 health workers; 3 FGDs with 24 patients	Late diagnosis/traditional therapies; staff shortage; inconsistent screening; patient confusion
Kusumo et al. [62]	Oct–Dec 2019	4 PHCs, Sleman, DIY	QE; TPA-based empowerment	N = 102 T2DM patients	Minimal knowledge gain; staff/logistics constraints

**Table 3.** Hindering factors identified in Prolanis implementation.

Thematic category	Examples of hindering factors
Infrastructure and staffing constraints (I/S)	Remote/transport barriers; limited space or clinic time for Prolanis; medication/lab-kit stock-outs; staff shortages and high workload; weak follow-up (no robust reminders, missed evaluations); poor coordination inside the clinic.
Low coverage, participation, and adherence (LCPA)	Medication nonadherence and program dropout; low family engagement/support.
Socioeconomic and cost barriers (S/C)	Transport/time-off/distance costs; drug affordability; limited internet coverage; facility budget constraints.
Cultural and health literacy barriers (C/HL)	Low health literacy/understanding of disease, treatment, and Prolanis benefits; low motivation/self-efficacy; preference for traditional remedies; stigma/fatalism; low community awareness; misconceptions about exercise and diet.
Pandemic-related disruptions (Pand)	Minimal two-way e-health (mostly only SMS reminders); limited phone/online follow-up during COVID-19; health service interruptions.

## 4. Discussion

For analytic consistency with global health guidance and our emphasis on local adaptation, we classified the five barrier themes against the WHO health-system building blocks. This framework aligns with Indonesia's health-system context [63]. From the 38 studies included in this review, it is evident that Prolanis faces multiple barriers that can be classified into five key categories, which reflect recurring themes and underlying issues that appear consistently throughout the literature, including infrastructure and staffing constraints (reported in 30 studies, 78.9%), low coverage and irregular participation (reported in 33 studies, 86.8%), socioeconomic and cost barriers (reported in 11 studies, 28.9%), cultural and health literacy gaps (reported in 18 studies, 47.4%), and pandemic-related disruptions (reported in 3 studies, 7.9%). A summary of key challenges in Prolanis implementation, aggregated from 38 studies, is presented in Table 4. Each category will be elaborated in the following subsections.

**Table 4.** Categorization of PROLANIS-implementation barriers reported in 38 studies.

**No.**	**Study (author, year of data collection)**	**I/S**	**LCPA**	**SE**	**C/HL**	**Pand**
1	Rahmawati, 2015 [36]	√		√		
2	Sulistyaningrum, 2016 [40]			√		
3	Mubarak, 2023 [26]	√				
4	Rokhmad, 2022 [37]	√	√	√	√	
5	Fajriani, 2024 [27]	√	√	√		
6	Iskandarsyah, 2018 [45]	√	√		√	
7	Aungsuroch, 2018 [29]		√	√	√	
8	Putri, 2017 [46]	√	√	√	√	
9	Tanjung, 2019 [28]	√	√	√		
10	Handayani, 2019–2020 [30]		√		√	
11	Khusna, 2018 [41]	√	√		√	
12	Azam, 2022 [43]		√	√		√
13	Paradise, 2023–2024 [47]	√	√			
14	Yusransyah, 2019 [48]	√	√		√	
15	Misnaniarti, 2018 [49]		√			
16	Armawati, 2018 [39]	√	√			
17	Alkaff, 2018–2019 [50]	√	√			
18	Sari, 2021 [24]	√	√		√	
19	Yudha, 2021 [51]				√	
20	Rofi'i, 2020 [42]	√			√	
21	Souhaly, 2020 [31]	√	√			
22	Zainuddin, 2022 [52]	√	√	√		
23	Soleman, 2019 [53]	√	√			
24	Sholihat, 2018 [54]				√	
25	Sholikatin, 2017 [55]		√		√	
26	Iskandarsyah, 2017–2018 [32]	√	√			
27	Salamah, 2019–2020 [44]	√	√			√
28	Krisnadewi, 2024 [33]	√	√	√	√	
29	Yuniartika, 2019 [56]	√	√		√	
30	Yusransyah, 2019 [38]	√	√			
31	Salamah, 2019–2020 [22]	√	√			√
32	Kusumawardana, 2023 [57]	√	√	√		
33	Rahmawati, 2016 [25]	√	√		√	
34	Febriawati, 2021–2022 [58]	√	√			
35	Alkaff, 2018–2019 [59]	√	√		√	
36	Said, 2023 [60]	√	√			
37	Sofyan, 2019–2020 [61]	√	√		√	
38	Kusumo, 2019 [62]	√	√		√	

Note: Abbreviation: I/S: infrastructure and staffing constraints; LCPA: low coverage, participation, and adherence; SE: socioeconomic and cost barriers; C/HL: cultural and health-literacy barriers; Pand: pandemic-related disruptions.

### 4.1. Infrastructure and staffing constraints

Infrastructure and staffing shortages map to WHO service delivery and health workforce domains, with spillover effects on access to essential medicines/diagnostics These shortages emerged as critical bottlenecks to Prolanis implementation, affecting the consistency and quality of patient services. Our synthesis indicates that delivery capacity is the foundational bottleneck. Multiple studies document staff shortages, role ambiguity, and supply interruptions in PHCs. In rural Yogyakarta, a survey of 384 patients reported shortfalls in clinical personnel, unclear nurse–doctor task division, medicine stock-outs, and multiple visits required to obtain refills [25]. In West Sulawesi, a qualitative assessment (n = 13 informants) found unequal human-resource allocation across PHCs and weak adherence to SOPs, leading to fragmented follow-up [26]. Facility-level constraints are recurrent: a South Sulawesi evaluation noted inconsistent availability of laboratory supplies, including HbA1c testing, limited space, and clashing schedules for Prolanis sessions [27]. Service quality signals point in the same direction, where audits across 25 PHCs identified the largest gaps in tangibility, empathy, and reliability [47]. Together, these data explain why structurally, Prolanis components often fail at the operational level, especially outside Java [33],[47],[50],[53],[58],[61],[62].

These observations mirror broader evidence from other LMICs, where gaps in physical infrastructure and human capital often limit the scope of community-based interventions [64]. A recent review by Nesengani et al. also found that understaffing and high workloads hindered comprehensive patient education and follow-up in rural PHCs [65], aligning with the heavy burden on Prolanis staff outlined by Mubarak et al. [26] and Rahmawati et al. [25]. Additionally, inadequate equipment, such as limited availability of HbA1c kits, has been consistently linked to suboptimal diabetes management, as reported by Klatman et al. in a multi-country study of LMICs [66]. Their findings underscore the importance of consistent funding to procure diagnostics and streamline laboratory services, echoing the challenges highlighted by Fajriani et al. [27]. To overcome these constraints, a recent study by Langlois et al. suggested targeted policy interventions and investments in health system strengthening [67]. As an example, structured capacity-building programs for both clinical and administrative PHC staff could alleviate workload pressures and clarify role divisions within multidisciplinary teams [68]. Furthermore, adopting technology-enabled solutions such as telemedicine may help bridge gaps in remote settings, although this approach requires parallel training and infrastructural support to ensure successful adoption [69]. In this regard, strengthening SOPs and establishing robust supply chains for essential materials have been deemed indispensable steps for sustaining program quality [70].

To restore capacity for planned, non-acute chronic care at the PHC level, physicians should institute protected Prolanis clinic sessions with standing order sets for biannual HbA1c or blood-pressure checks and formally escalate stock-out events with contingency pathways for laboratories and medicines. Nurses should maintain a patient registry with recall, run weekly missed-visit reports, and complete pre-visit planning, while coordinating space and session logistics to avoid clashes reported in several PHCs [44]. Pharmacists can stabilize operations by deploying stock-monitoring dashboards, synchronizing refills, and delivering brief adherence counselling at pickup when staffing permits [38],[47]. Program managers should set minimum staffing norms for Prolanis sessions, and formalize SOPs for patient flow, laboratories, and referrals [22],[44]. They should also adopt supply-chain KPIs with routine review. Monitoring indicators include the proportion with HbA1c documented within six months, stock-out days for essential items, and the share of sessions conducted as scheduled.

### 4.2. Low coverage, participation, and adherence

Another recurring obstacle to the implementation of Prolanis is related to the limited coverage and irregular participation of enrolled patients. Competing commitments, travel barriers, and lack of perceived benefits were cited by many participants as the reason not to participate in Prolanis. Engagement shortfalls are widespread and quantifiable. In three East-Java PHCs, only 187 of 546 people with T2DM (34.3%) were enrolled in Prolanis, which was attributed by the authors to transport barriers and limited outreach [37]. Even when patients do enroll, dropout rates remain substantial. Misnaniarti et al. reported that only 56.8% of individuals continued attending Prolanis sessions, despite evidence of improved quality of life among regular attendees [49]. This trend aligns with Sholikatin et al., who found that roughly half of participants in their study were non-adherent, citing work obligations and family responsibilities as reasons for missing group meetings [55]. Moreover, Iskandarsyah et al. observed that many patients simply collect their medications without partaking in group educational or support activities, indicating a lack of perceived benefit of Prolanis beyond medication access [45],[50].

These challenges resonate with findings from other LMICs, where community-based interventions often struggle with suboptimal patient retention and inconsistent attendance [71]. A systematic review by Rosen et al. similarly documented high attrition rates in sub-Saharan Africa, attributing them to low perceived program utility and insufficient follow-up mechanisms [72], factors that parallel the Prolanis experience [45]. In Iran, Sharifi et al. found that participants dropped out of diabetes self-management groups when immediate health gains were not evident [73], aligning with the perception that Prolanis offers limited short-term benefit [49].

To address these issues, a recent study by Aschbrenner et al. highlighted the value of peer support networks and motivational counselling, which may heighten patients' engagement and perceived relevance of group activities [74]. Such measures could be particularly beneficial in the Indonesian context, where cultural norms often emphasize familial and communal support [55].

To improve reach and retention, physicians should risk-stratify patients with uncontrolled HbA1c or blood pressure and set personalized follow-up intervals, simplifying regimens where possible to reduce visit burden [23]. Nurses should replace one-way SMS with two-way reminders or social messaging apps, offer after-hours or rotating sessions, and perform defaulter tracing within 48 hours [23],[46], while facilitating peer-support or coach-led groups to increase perceived relevance. Pharmacists can synchronize refills to a single monthly pickup, provide blister packs or dosing calendars, and send due-refill prompts. Program managers should pilot transport vouchers for hard-to-reach villages [23],[39], while maintaining retention dashboards (attendance %, defaulter rate) at the PHC level. Key indicators are enrolment among eligibles, attendance and retention ≥70%, recall success rate, and proportion using two-way messaging.

### 4.3. Socioeconomic and cost barriers

Socioeconomic and cost barriers align with WHO financing and access to essential medicines/diagnostics and remain a prominent challenge to the success of Prolanis, often limiting patients' ability to consistently access medications and attend scheduled sessions [57]. Across 16 PHCs in Yogyakarta (n = 293), per-prescription costs were higher in T2DM or T2DM + hypertension, stressing PHC budgets and patients alike [40]. Tanjung et al. reported that variability in transport and consultation fees hinders uniform adoption of Prolanis, particularly in remote areas where travel costs may exceed household budgets [28]. Compounding these problems, Rahmawati et al. documented that approximately 17% of patients resort to purchasing over-the-counter drugs, either because of insufficient finances or inconsistent medication supplies, consequently undermining proper disease management [36].

Similar findings are also found within broader research in LMICs, where out-of-pocket expenses are consistently cited as a primary barrier to sustained engagement in community-based interventions [75]. A multi-site study in sub-Saharan Africa by Kabia et al. revealed that patients frequently compromise on essential therapies due to competing household expenses [76], mirroring the financial pressures observed in Indonesian Prolanis participants [40].

In rural Malawi, Wang et al. found that sporadic transport availability and prohibitive travel costs undermined the continuity of hypertension control programs [77], an issue that parallels the transport-related barriers emphasized by Tanjung et al. [28]. Additionally, limited public funding for health education materials often exacerbates inequities in patient knowledge, leading to reduced adherence and delayed care-seeking [78].

Addressing these socioeconomic and cost hurdles requires a multifaceted approach. Community-level microfinance or social insurance schemes could expand access to regular check-ups and diagnostic services [79]. Furthermore, incorporating telemedicine tools can help reduce transport costs and mitigate the effects of uneven geographical coverage, although it must be accompanied by digital literacy programs to ensure equitable access [69].

To reduce friction costs that depress participation and testing, physicians should prioritize cost-effective generics, implement multi-month dispensing for stable patients, and avoid non-essential laboratory tests [28],[37]. Nurses ought to screen for transport or financial constraints during intake and cluster appointments so that laboratory testing, consultation, and refill occur in one trip. Pharmacists should provide transparent cost options and generic substitution, reconcile polypharmacy, and check for herb–drug interactions given local use patterns [22],[29]. Program managers can plan budgets for diagnostics, streamline claims for Prolanis tests, and explore local transport support agreements. Suggested metrics include the proportion of multi-month dispensing, documented out-of-pocket red flags, and the quarterly laboratory completion rate.

### 4.4. Cultural and health literacy barriers

Cultural and health literacy barriers correspond to WHO's “service delivery” and “information”, emphasizing patient capability and acceptability. In many Indonesian communities, ingrained cultural beliefs and limited health literacy impede optimal chronic disease management through Prolanis [46]. For instance, in Belitung (n = 20), participants reported a preference for traditional or herbal remedies, self-medication, dosing confusion, and transport obstacles to clinic visits [29]. Likewise, Handayani et al. documented unhealthy dietary patterns and limited patient education, with differences between Java and non-Java settings [30]. Even where patients acknowledge the need for change, Khusna et al. found notable gaps in nutrition knowledge and a dearth of consistent counselling, leading to sporadic adherence to recommended diets [41]. Further compounding these issues, Yuniartika et al. highlighted that educational materials often follow a uniform approach, failing to address diverse cultural norms, varying literacy levels, and unique learning styles, particularly in rural or low-resource settings [56]. Psychosocial needs are present but under-addressed. A Bali survey (n = 384) found mild anxiety common among Prolanis participants [51],[54].

Similar challenges have been noted in other LMICs. A cross-sectional study in sub-Saharan Africa by Macquart de Terline et al. revealed that localized dietary habits that are often high in salt and reliant on traditional herb-based treatments could significantly hinder the uptake of community-based hypertension programs [80]. Parallel findings emerged from a multi-regional investigation in South Asia, where Patel et al. observed that limited health literacy and prevailing cultural beliefs about natural medicine created persistent distrust of clinical therapies [81]. Addressing such barriers necessitates tailored educational strategies, including culturally adapted training modules and interactive, community-driven demonstrations [82]. Moreover, Mistry et al. emphasized the role of peer support groups that integrate local languages and examples, thus enhancing both comprehension and acceptability of evidence-based medical guidance [83].

To raise acceptability and self-efficacy, physicians should employ teach-back, co-produce one-page care plans, and discuss traditional remedies respectfully, addressing potential interactions [29],[45]. Brief screening for anxiety or depression with referral when positive is advised [41]. Nurses should deliver culturally adapted education (local foods, plate models), include family members in counselling, and lead walking groups. Pharmacists can provide dose-timing aids (calendars, pictograms), conduct medication reviews with herb–drug counselling, and reinforce action plans. Program managers should develop local-language materials, train community cadres, and schedule patients' feedback on education quality [30],[49].

### 4.5. Pandemic-related disruptions

The COVID-19 pandemic introduced significant disturbances to Prolanis' implementation in Indonesia, particularly because of total mobility restrictions and the rapid redirection of healthcare resources [31],[52]. In Central Java (n = 164), movement restrictions led to missed visits and interrupted medication, with higher fasting glucose and HbA1c during the restriction period [43]. Similarly, East-Java cohorts (n = 91; n = 52) reported fewer follow-ups due to staff redeployment, limited screening, and ad-hoc telemedicine [22],[44]. In parallel, Fajriani et al. noted that digital outreach programs were hindered by the lack of smartphone access for some participants, restricting the effectiveness of telemedicine or messaging-based appointment reminders [27].

These challenges align with emerging evidence across LMICs, where community-based interventions have been disproportionately affected by the pandemic. A study by Coates et al. revealed that public health emergencies often lead to the diversion of healthcare personnel to acute care roles [84], like the staff reassignments observed by Salamah et al. [44]. This reallocation decreases the capacity for chronic disease management, especially in rural or remote clinics with limited backup personnel [84]. Furthermore, Mbunge et al. found that telemedicine adoption in resource-constrained settings faces substantial barriers, including inadequate internet connectivity, limited device ownership, and low digital literacy [85]. These constraints echo Fajriani et al.'s findings of insufficient smartphone use for Prolanis reminders [27]. Additionally, patients who rely on public transportation or must travel long distances for follow-ups, challenges noted in Azam et al. [43], were especially vulnerable when lockdown measures severely restricted mobility [86].

To mitigate these disruptions, some programs in LMICs have experimented with blending in-person visits with remote consultations to maintain medication adherence and patient education [87]. However, such adaptations require deliberate infrastructure investments, including robust telehealth platforms, clear clinical pathways, and ongoing training for healthcare workers [88]. By incorporating these strategies, healthcare systems may better prepare for future public health crises while safeguarding the long-term management of chronic conditions like hypertension and diabetes.

To protect continuity during shocks, physicians should define a minimum care bundle, such as multi-month prescriptions and an HbA1c testing window, and escalate hyperglycemia promptly [15],[32],[33]. Nurses should operate a triage phone or social messaging app line, coach home blood pressure or glucose logs, and stagger appointments to maintain distancing. Pharmacists can offer delivery of pre-packed refills and remote counselling. Program managers should maintain buffer stocks of medicines, approve hybrid-care SOPs and task-shifting during emergencies, and ensure PPE and risk communication. Continuity indicators include the share of multi-month dispensing during emergencies, missed-visit recovery within 7 days, and maintenance of HbA1c testing rates within the target window.

## 5. Conclusions

The findings from these studies collectively suggest that while Prolanis holds considerable potential as a chronic disease management strategy in Indonesia, its implementation remains challenged by a range of structural, cultural, and operational barriers. These include infrastructure and staffing constraints, low coverage, participation and adherence, socioeconomic and cost barriers, cultural and health literacy barriers, and pandemic-related disruptions.

Addressing these multifaceted challenges is essential to optimizing the reach and impact of Prolanis. Investment in the health system, stronger community outreach strategies, and development of clear SOPs could significantly enhance program effectiveness. Moreover, peer support groups that integrate local languages and motivational counselling, as well as community-level microfinance or social insurance schemes, are critical to sustaining long-term participation and improving health outcomes for individuals living with NCD.

Emerging digital health technologies, such as telemedicine, e-health, and m-health, offer promising avenues for overcoming logistical and geographical barriers. E-health platforms can help extend access to care in underserved and remote regions, reduce patient transportation costs, and enable more continuous monitoring and communication between patients and providers. However, the successful integration of such technologies requires parallel investments in digital infrastructure, healthcare provider training, and digital literacy programs for patients. Thus, a comprehensive and context-sensitive approach is needed to ensure that digital solutions complement and strengthen the existing Prolanis framework.

Although the information gathered was extensive, some limitations need to be considered in interpreting this review. First, the search strategy was limited to three major databases (Scopus, ScienceDirect, and PubMed). As a result, language and indexing bias may have been introduced, and locally published studies may be under-represented. Second, this review excludes grey literature, such as government reports or policy briefs, which could potentially omit operational data critical to understanding real-world implementation challenges. Lastly, the heterogeneity of study settings and patient populations, while reflecting the diversity of Indonesia, limits the potential for standardized comparisons. As Indonesia's healthcare system continues to evolve, the findings presented here may also change over time, especially as new policy changes reshape the way Prolanis is delivered.

## Use of AI tools declaration

The authors declare they have not used Artificial Intelligence (AI) tools in the creation of this article.


