# Supplementary material for: Challenges in implementing Indonesia's community-based chronic disease management program (Prolanis): A scoping review
Source: AIMS Public Health. 2025 Sep 11;12(3):890–915. doi: 10.3934/publichealth.2025045 (PMC12538241; doi:10.3934/publichealth.2025045)
Supplement: Supplementary file 1 [file publichealth-12-03-045-s001.pdf]

Public Health

AIMS Public Health, 12(3): 890–915.
DOI: 10.3934/publichealth.2025045
Received: 09 July 2025
Revised: 17 August 2025
Accepted: 02 September 2025
Published: 11 September 2025

https://www.aimspress.com/journal/aimsph

Review

Challenges in implementing Indonesia's community-based chronic disease management program (Prolanis): A scoping review

Raden M. Febriyanti[1,*], Aalbrecht Alby Irawan[2], Nursanti Anggriani[3] , Yudhie Andriyana[4] and Rizky Abdulah[5]

[1] Department of Biological Pharmacy, Faculty of Pharmacy, Universitas Padjadjaran, Jatinangor, West Java, Indonesia
[2] Doctoral Program in Mathematics, Faculty of Mathematics and Natural Science, Universitas Padjadjaran, Jatinangor, West Java, Indonesia
[3] Department of Mathematics, Faculty of Mathematics and Natural Science, Universitas Padjadjaran, Jatinangor, West Java, Indonesia
[4] Department of Statistics, Faculty of Mathematics and Natural Science, Universitas Padjadjaran, Jatinangor, West Java, Indonesia
[5] Department of Pharmacology and Clinical Pharmacy, Faculty of Pharmacy, Universitas Padjadjaran, Jatinangor, West Java, Indonesia

* **Correspondence:** Email: raden.maya@unpad.ac.id.

Supplementary

File S1. Full database search strategies.

Scope

- Topic: Barriers to implementation of Indonesia's Program Pengelolaan Penyakit Kronis (Prolanis)
- Population/setting: Primary health care (puskesmas/PHC) in Indonesia
- Study types: Peer-reviewed journal articles. Grey literature excluded.
- Years covered: 2014–2024

- Language: No language limits applied at the search stage. Eligibility allowed English and Indonesian full texts.
- De-duplication: All records exported to Mendeley Reference Manager. Duplicates removed prior to screening.

A. PubMed

Interface: https://pubmed.ncbi.nlm.nih.gov/
Line-by-line strategy (field tags in brackets):
#1 PROLANIS[Title/Abstract]
#2 "Program Pengelolaan Penyakit Kronis" [Title/Abstract]
#3 "Program Pengelolaan Penyakit Kronik" [Title/Abstract]
#4 #1 OR #2 OR #3
#5 Filters applied in the interface: Publication dates from 2014/01/01 to 2024/12/31; Species: Humans; Article types: Journal Article, Clinical Trial, Observational Study, Quasi-Experimental Study, Evaluation Study; No language filter.
Copy/pasteable single-string equivalent:
("PROLANIS" [tiab] OR "Program Pengelolaan Penyakit Kronis" [tiab] OR "Program Pengelolaan Penyakit Kronik" [tiab]) AND ("2014/01/01" [Date-Publication]: "2024/12/31" [Date-Publication]) AND Humans [Mesh] AND (Journal Article [ptyp] OR Clinical Trial [ptyp] OR Observational Study [ptyp] OR Quasi-Experimental Study [ptyp] OR Evaluation Study [ptyp])

B. Scopus (Elsevier)

Interface: https://www.scopus.com|Final run: 30 June 2024
Search field: TITLE-ABS-KEY
TITLE-ABS-KEY(PROLANIS OR "Program Pengelolaan Penyakit Kronis" OR "Program Pengelolaan Penyakit Kronik") AND (PUBYEAR > 2013 AND PUBYEAR < 2025)
Filters: Document type = Article OR Review; Subject area = Medicine OR Nursing OR Health Professions OR Social Sciences (Health-related); Language = All; Source type = Journals.

C. ScienceDirect (Elsevier)

Interface: https://www.sciencedirect.com|Final run: 30 June 2024
Advanced search (All fields):
("PROLANIS" OR "Program Pengelolaan Penyakit Kronis" OR "Program Pengelolaan Penyakit Kronik")
Filters: Years = 2014–2024; Article type = Research articles and Reviews (journals only); Subject areas = Medicine; Nursing; Health Professions; Public Health and Health Policy; Language = All.

D. Handsearching and forward–backward citation chasing

- Reference lists of all included studies were screened.
- Cited-by searches were performed in Scopus for sentinel Prolanis articles to identify recent evaluations and implementation studies.

Export and record management

- All results were exported in RIS/CSV formats and imported into Mendeley Reference Manager.
- De-duplication was run automatically and verified manually prior to screening.
- Counts by database and disposition are summarised in the PRISMA flow diagram in the manuscript.

File S2. Preferred Reporting Items for Systematic reviews and Meta-Analyses extension for Scoping Reviews (PRISMA-ScR) Checklist.

SECTION	ITEM	PRISMA-ScR CHECKLIST ITEM	REPORTED ON PAGE #
**TITLE**			
Title	1	Identify the report as a scoping review.<br><br>This manuscript is explicitly titled "Challenges in implementing Indonesia's community-based chronic disease management program (Prolanis): A scoping review".	1
**ABSTRACT**			
Structured summary	2	Provide a structured summary that includes (as applicable) background, objectives, eligibility criteria, sources of evidence, charting methods, results, and conclusions that relate to the review questions and objectives.<br><br>A structured abstract addressing these elements—background, objectives, eligibility criteria, sources of evidence, charting methods, main findings, and conclusions.	1–2
**INTRODUCTION**			
Rationale	3	Describe the rationale for the review in the context of what is already known. Explain why the review questions/objectives lend themselves to a scoping review approach.<br><br>The rationale is provided on **pages 2–4**, where the manuscript discusses existing knowledge on non-communicable diseases (NCDs) in low- and middle-income countries and why Prolanis, as a community-based program, faces unique barriers in Indonesia. These pages also explain how the breadth of evidence and the program's diverse contexts warrant a scoping review rather than a narrower systematic review.	2–4
Objectives	4	Provide an explicit statement of the questions and objectives being addressed with reference to their key elements (e.g., population or participants, concepts, and context) or other relevant key elements used to conceptualize the review questions and/or objectives.<br><br>The manuscript explicitly states on **page 3** that its objectives	3

		are to examine Prolanis barriers and facilitators across multiple Indonesian regions (context), focusing on individuals with chronic illnesses (population) in community-based interventions (concept). Five core research questions frame the temporal scope, geographical settings, study methodologies, quantitative findings, and key factors that hinder or facilitate Prolanis performance.	
**METHODS**			
Protocol and registration	5	Indicate whether a review protocol exists; state if and where it can be accessed (e.g., a Web address); and if available, provide registration information, including the registration number.<br><br>On **page 4**, the manuscript clarifies that no prospective registration or publicly accessible protocol was created for this scoping review.	4
Eligibility criteria	6	Specify characteristics of the sources of evidence used as eligibility criteria (e.g., years considered, language, and publication status), and provide a rationale.<br>The eligibility criteria are described on **pages 4**, stating that studies must be peer-reviewed, published between 2014 (Prolanis' inception year) and 2024, and include primary data or descriptive analyses related to Prolanis implementation.	4
Information sources*	7	Describe all information sources in the search (e.g., databases with dates of coverage and contact with authors to identify additional sources), as well as the date the most recent search was executed.<br><br>On **page 4**, it is explained that three major bibliographic databases—Scopus, ScienceDirect, and PubMed—were searched from 2014 through 2024.	4
Search	8	Present the full electronic search strategy for at least 1 database, including any limits used, such that it could be repeated.<br>The manuscript (on **page 4**) provides the main search string for PubMed, which combined terms for Prolanis. Similar search adaptations were applied to Scopus and ScienceDirect.	4
Selection of sources of evidence†	9	State the process for selecting sources of evidence (i.e., screening and eligibility) included in the scoping review.<br><br>On **page 4**, the manuscript describes how titles and abstracts were screened by two independent reviewers to remove irrelevant articles. Potentially eligible full texts were then assessed against inclusion and exclusion criteria, with disagreements resolved by consensus.	4
Data charting process‡	10	Describe the methods of charting data from the included sources of evidence (e.g., calibrated forms or forms that have been tested	4

		by the team before their use, and whether data charting was done independently or in duplicate) and any processes for obtaining and confirming data from investigators.<br><br>On **page 4**, it is noted that a standardized data extraction form was used. Two reviewers independently extracted study details (authors, year, location, methodology, key findings), resolving discrepancies by discussion.	
Data items	11	List and define all variables for which data were sought and any assumptions and simplifications made.<br><br>The manuscript, on **page 5**, specifies that the variables of interest included publication details, geographic context, study design, Prolanis components examined, and identified barriers or facilitators. Any assumptions, such as grouping similar challenges under broader categories, are explicitly noted in the data-charting files.	5
Critical appraisal of individual sources of evidence§	12	If done, provide a rationale for conducting a critical appraisal of included sources of evidence; describe the methods used and how this information was used in any data synthesis (if appropriate).<br><br>On **page 4**, it is stated that no formal critical appraisal tool was used, as the primary goal was to map the range of evidence.	4
Synthesis of results	13	Describe the methods of handling and summarizing the data that were charted.<br><br>The manuscript notes on **page 4** that descriptive analyses of study attributes and inductive thematic analysis of Prolanis barriers/facilitators were performed to identify recurring patterns. Discrepancies were resolved by discussion among the review team, and new categories were created as needed when similar barriers emerged under slightly different terms.	4
**RESULTS**			
Selection of sources of evidence	14	Give numbers of sources of evidence screened, assessed for eligibility, and included in the review, with reasons for exclusions at each stage, ideally using a flow diagram.<br><br>On **page 5**, the manuscript indicates that 81 records were identified, 18 duplicates removed, 63 full-text articles assessed, and 25 excluded for not meeting inclusion criteria. Ultimately, 38 studies were included in the review. Figure 1 depicts the corresponding PRISMA flow diagram.	5
Characteristics of sources of	15	For each source of evidence, present characteristics for which data were charted and provide the citations.	9–19

evidence		On **pages 9–19** and in Table 2, the manuscript presents the study characteristics (author, year, location, design, sample, and main findings), with full citations linked to each entry.	
Critical appraisal within sources of evidence	16	If done, present data on critical appraisal of included sources of evidence (see item 12).<br><br>As noted under Item 12 and re-stated on **page 4**, no formal appraisal was conducted. Any limitations the original authors reported (e.g., small sample size) are mentioned in Table 3 to provide context.	4
Results of individual sources of evidence	17	For each included source of evidence, present the relevant data that was charted that relates to the review questions and objectives.<br><br>Table 2 provides an overview of each study's key data, including Prolanis outcomes, measured barriers, and proposed facilitators for better implementation.	9–19
Synthesis of results	18	Summarize and/or present the charting results as they relate to the review questions and objectives.<br><br>On **pages 20**, the manuscript synthesizes results by describing the recurring themes: infrastructure shortages, socioeconomic obstacles, cultural barriers, patient adherence issues, and pandemic-related disruptions. These collective findings directly address the five overarching research questions posed in the introduction.	20
**DISCUSSION**			
Summary of evidence	19	Summarize the main results (including an overview of concepts, themes, and types of evidence available), link to the review questions and objectives, and consider the relevance to key groups.<br><br>On **pages 21–28**, the main results are contextualized within the broader literature, highlighting that Prolanis faces multi-level barriers yet shows promise for chronic disease management in Indonesia. The discussion explicitly relates these findings to the initial review objectives and highlights key stakeholder groups (e.g., policymakers, healthcare providers, patients) who may benefit from the conclusions.	21–28
Limitations	20	Discuss the limitations of the scoping review process.<br><br>On **pages 27**, the manuscript acknowledges limitations, including restricting searches in three databases. Heterogeneity in study design and setting also constrained standardized comparisons.	27

Conclusions	21	Provide a general interpretation of the results with respect to the review questions and objectives, as well as potential implications and/or next steps.<br><br>The manuscript's conclusion on **page 27** explains that Prolanis can be strengthened by addressing patient, system, infrastructure, and sociocultural barriers, with implications for refining chronic disease programs across Indonesia and potentially other low- and middle-income countries.	27
**FUNDING**			
Funding	22	Describe sources of funding for the included sources of evidence, as well as sources of funding for the scoping review. Describe the role of the funders of the scoping review.<br><br>On **page 27** under "Acknowledgments," the manuscript states that the Rector of Universitas Padjadjaran funded the Article Processing Charges through the Directorate of Research and Community Engagement. No specific funding source influenced the design or conduct of this scoping review, and the sponsors played no role in data collection, analysis, or interpretation.	27

Note: JBI = Joanna Briggs Institute; PRISMA-ScR = Preferred Reporting Items for Systematic reviews and Meta-Analyses extension for Scoping Reviews. *Where *sources of evidence* (see second footnote) are compiled from, such as bibliographic databases, social media platforms, and Web sites. †A more inclusive/heterogeneous term used to account for the different types of evidence or data sources (e.g., quantitative and/or qualitative research, expert opinion, and policy documents) that may be eligible in a scoping review as opposed to only studies. This is not to be confused with *information sources* (see first footnote). ‡The frameworks by Arksey and O'Malley (6) and Levac and colleagues (7) and the JBI guidance (4, 5) refer to the process of data extraction in a scoping review as data charting. §The process of systematically examining research evidence to assess its validity, results, and relevance before using it to inform a decision. This term is used for items 12 and 19 instead of "risk of bias" (which is more applicable to systematic reviews of interventions) to include and acknowledge the various sources of evidence that may be used in a scoping review (e.g., quantitative and/or qualitative research, expert opinion, and policy document).

St. Michael's
Inspired Care.
Inspiring Science.

From: Tricco AC, Lillie E, Zarin W, et al. (2018) PRISMA Extension for Scoping Reviews (PRISMAScR): Checklist and Explanation. *Ann Intern Med* 169: 467–473. https://doi.org/10.7326/M18-0850

AIMS Press

© 2025 the Author(s), licensee AIMS Press. This is an open access article distributed under the terms of the Creative Commons Attribution License (https://creativecommons.org/licenses/by/4.0)
